# Identification and Functional Characterization of a Novel POU3F4 Frameshift Mutation in a Chinese Family

**DOI:** 10.3390/life16060868

**Published:** 2026-05-22

**Authors:** Shuwen Fan, Yaqiong Guan, Mengya Xiang, Hongzhe Yu, Tianyang Zhang, Jialei Fu, Jiahao Fei, Yongtao Xiao, Yunfeng Wang

**Affiliations:** 1 ENT Institute, Department of Otorhinolaryngology, EYE & ENT Hospital, Fudan University, Shanghai 200031, China; 24111260027@m.fudan.edu.cn (S.F.); 20211260023@fudan.edu.cn (M.X.); 25111260044@m.fudan.edu.cn (H.Y.); tianyangzhang25@m.fudan.edu.cn (T.Z.); 23211260033@m.fudan.edu.cn (J.F.); 2 NHC Key Laboratory of Hearing Medicine, Fudan University, Shanghai 200031, China; 3 Department of Medical Technology and Information Engineering, Zhejiang Chinese Medical University, Hangzhou 310053, China; guanyaqiong1212@163.com (Y.G.); 202511114411003@zcmu.edu.cn (J.F.)

**Keywords:** *POU3F4* gene, X-linked deafness 2, genetics of hearing loss, Frameshift mutation

## Abstract

Hereditary sensorineural hearing loss (SNHL) represents a significant global public health burden. DFNX2, an X-linked form of non-syndromic SNHL, is caused by pathogenic variants in the *POU3F4* gene. This study aimed to identify a novel *POU3F4* mutation and characterize its functional consequences to elucidate the molecular pathogenesis of DFNX2. A three-generation Chinese family with X-linked deafness was recruited. Targeted next-generation sequencing was used to screen candidate variants, which were validated by Sanger sequencing for co-segregation analysis. Functional assays, including subcellular localization, dual-luciferase reporter assay, Western blotting, and homology modeling, were performed to assess the mutation’s effects. A novel frameshift mutation, c.670_673dupGGTA (p.(Asn225Argfs*2)), was identified and showed complete co-segregation with the deafness phenotype. The mutant protein exhibited cytoplasmic mislocalization, and dual-luciferase assays revealed a severe reduction in transcriptional activation capacity, whereas Western blot confirmed stable expression of the truncated protein. Structural modeling predicted the loss of both the POUS and POUH DNA-binding subdomains. Collectively, this study expands the mutational spectrum of *POU3F4* and supports previously reported mechanisms underlying DFNX2 pathogenesis.

## 1. Introduction

Hereditary sensorineural hearing loss (SNHL), with an incidence of 1–3 per 1000 live births, represents a significant public health challenge. Most hereditary deafness cases result in profound congenital or early-onset deafness that critically impairs speech development, learning, and educational outcomes [1,2,3]. Over recent decades, efforts to decipher the genetic architecture of human SNHL have yielded profound insights into the molecular mechanisms governing auditory system development and function, identifying hundreds of genes implicated in monogenic hearing loss [4].

Approximately 70% of hereditary deafness cases are non-syndromic hearing loss (NSHL). While most causative defects are autosomal, 1–5% of cases result from mutations in X-chromosomal genes [5,6]. To date, seven distinct X-linked deafness loci (DFNX1–DFNX7) have been mapped, with four corresponding genes definitively identified: *PRPS1* (DFNX1) [7], *POU3F4* (DFNX2) [8], *SMPX* (DFNX4) [9,10] and *AIFM1* (DFNX5) [11] (https://hereditaryhearingloss.org/, accessed 30 June 2025).

DFNX2 is the most prevalent form of X-linked NSHL [8,12]. It is clinically characterized by mixed, progressive conductive and sensorineural hearing loss, often profound from early childhood, accompanied by distinctive temporal bone malformations. Key radiographic findings include dilatation of the internal auditory canal (IAC) and aberrant communication (fistula) between the IAC and the basal turn of the cochlea. This anomaly poses a significant surgical risk, as stapedectomy can lead to a perilymphatic “gusher” [12,13].

DFNX2 is caused by pathogenic variants in the *POU3F4* gene (NM_000307.3, POU Class 3 Homeobox 4), which encodes a transcription factor of the POU superfamily, subclass III. POU3F4 functions as a sequence-specific DNA-binding protein that recognizes the octamer motif (ATGCAAAT) to regulate target genes essential for inner ear development [14,15]. POU3F4 is expressed in the otic mesenchyme during embryogenesis, where it promotes spiral ganglion neuron survival and stimulates axon extension toward hair cells [16]. Furthermore, *POU3F4*-deficient mice exhibit abnormalities in specialized fibrocytes distinct from the bony cochlear wall, a marked reduction in endocochlear potential, and profound hearing loss, indicating that loss of POU3F4 disrupts cochlear fluid homeostasis [17,18,19]. At the cellular level, *POU3F4* mutations may lead to cochlear cell damage and dysfunction by modulating mitochondrial function, calcium concentration, and apoptosis [20].

Given the severe phenotypic consequences of *POU3F4* mutations and its fundamental roles in cochlear development, the identification and functional characterization of novel pathogenic variants remain important for understanding the molecular pathogenesis of DFNX2 and refining genotype–phenotype correlations. This study reports the identification and functional characterization of a *POU3F4* frameshift variant (c.670_673dupGGTA, p.(Asn225Argfs*2)) in a patient with classic DFNX2 features. This variant is novel and has not been previously documented in the ClinVar, dbSNP, or gnomAD database.

## 2. Materials and Methods

### 2.1. Subjects and Clinical Evaluation

A three-generation Chinese family with two male affected individuals presenting X-linked nonsyndromic hearing loss was enrolled after obtaining written informed consent. Evaluation included: (1) detailed family history collection with documentation of hearing loss onset, progression, and associated symptoms; (2) otoscopic examination; (3) pure-tone audiometry; and (4) high-resolution temporal bone CT imaging.

### 2.2. Mutation Analysis

Genomic DNA was extracted from peripheral blood and subjected to a targeted next-generation sequencing (NGS) covering 127 deafness-related genes (Beijing Genomics Institute). Sequencing was performed on the BGI-SEQ500 platform. The target region size was 496,051 bp, with a mean sequencing depth of 165.56×; 99.77% of target bases were covered at ≥30×, and the overall target region coverage was 99.96%. Post-sequencing, clean reads were aligned to the human reference genome (hg19) using the Burrows-Wheeler Aligner (BWA). All variants were further filtered and annotated using multiple databases, including the National Center for Biotechnology Information (NCBI), 1000 Genomes, dbSNP, and HGMD. This method is not designed for heterozygous large-fragment copy number variations (CNV), complex rearrangements, or dynamic mutations. The list of the 127 genes is provided in Supplementary Table S1. Bioinformatic analysis was conducted to filter and prioritize candidate variants, followed by Sanger sequencing for variant validation and co-segregation analysis across the family. Sanger sequencing was performed using specific primers flanking the POU3F4 variant (NM_000307.3) on the proband and all available family members to confirm the variant and its co-segregation with the phenotype. The pathogenicity of the identified variant was assessed according to the American College of Medical Genetics and Genomics (ACMG) guidelines.

### 2.3. Cell Culture

293T cells (Cat No.FH0244, provided by Shanghai Fuheng Biotechnology Co., Ltd., Shanghai, China) were cultured in Dulbecco’s Modified Eagle Medium (DMEM) supplemented with 10% fetal bovine serum (FBS) and 1% penicillin–streptomycin (100 U/mL penicillin and 100 µg/mL streptomycin) at 37 °C in a sterile environment containing 5% CO_2_. Cells were passaged at 80–90% confluence.

### 2.4. Plasmid Construction

Recombinant plasmids encoding human wild-type (WT) *POU3F4* and mutant *POU3F4* (c.670_673dupGGTA, p.Asn225Argfs*2) were constructed using standard cloning methods. The expression vector *pCDNA3.1-CMV-MCS-EF1-ZsGreen-T2A-puro* (Hanbio Biotechnology, Shanghai, China) was linearized by double digestion with *EcoRI* and *BamHI* (Thermo Fisher Scientific, Shanghai, China) and gel-purified. The WT *POU3F4* open reading frame was PCR-amplified using specific primers. The c.670_673dupGGTA mutant was generated by overlap-extension PCR with three primer pairs (Supplementary Table S2).

Purified inserts and linearized vector were ligated using the HB-infusion™ HD Cloning Kit (Hanbio Biotechnology, Shanghai, China), transformed into *E. coli* DH5α, and plated on ampicillin–LB agar. Positive clones were screened by colony PCR, with two clones per construct Sanger-sequenced for validation. Plasmids were purified to meet quality standards (OD 260/280 = 1.8–2.0; concentration > 200 ng/μL).

### 2.5. Transient Transfection and Immunofluorescence Analysis

293T cells were seeded in 24-well plates 24 h prior to transfection. At 70% confluency, cells were transfected with control, wild-type or mutant *POU3F4*-*3xFlag* plasmids using Lipofectamine 3000 (Thermo Fisher Scientific, Shanghai, China) according to the manufacturer’s protocol. After 24h incubation, cells were fixed with 4% paraformaldehyde for 15 min at room temperature, followed by permeabilization and blocking in PBS containing 0.2% Triton X-100 and 10% donkey serum for 1 h.

Primary antibody incubation was performed overnight at 4 °C using monoclonal mouse anti-FLAG antibody (1:1000, Abmart, Shanghai, China). After PBS washes, samples were incubated with Alexa Fluor 555-conjugated donkey anti-mouse secondary antibody (1:500, Invitrogen, Waltham, MA, USA) for 1 h at room temperature. Nuclei were counterstained with DAPI (1:800 dilution, 10 min). Imaging was conducted using a Zeiss laser-scanning confocal microscope with a 63× oil immersion objective.

For quantitative analysis, 100 transfected cells per condition were randomly selected from three independent experiments. Subcellular localization was assessed by calculating the percentage of cells exhibiting cytoplasmic POU3F4 signal.

### 2.6. Dual-Luciferase Assay

Transcriptional auto-regulation of POU3F4 was assessed using a dual-luciferase reporter system in 293T cells. The promoter region (−482 to +25 bp relative to transcription start site) was cloned into the *pGL3*-basic vector to construct the reporter plasmid [21]. For transfection, cells seeded in 24-well plates at 70% confluency received a mixture of 250 ng reporter plasmid, 250 ng effector plasmid (WT or mutant *POU3F4-3xFlag*), and 25 ng *pRL-TK* normalization plasmid, combined with liposomal transfection reagent (Genomeditech, Shanghai, China) in 50 µL Opti-MEM (Gibco, Shanghai, China). After 15 min incubation, complexes were added to cells and incubated for 48 h. Luciferase activity was measured using a dual-luciferase assay kit (Genomeditech, Shanghai, China) 48 h after transfection, with results expressed as Firefly/Renilla RLU ratios normalized to empty vector controls. Data represent the mean of three independent experiments.

### 2.7. Western Blotting

To evaluate the expression levels of WT and mutant POU3F4-3xFlag proteins, Western blot analysis was performed. 293T cells were seeded in 6-well plates and transfected with empty vector, WT or mutant POU3F4 plasmids using Lipofectamine 3000. After 48 h, cells were harvested and lysed in RIPA buffer supplemented with protease inhibitors. Equal amounts of protein were separated by 10% SDS-PAGE and transferred onto PVDF membranes. Membranes were blocked with 5% non-fat milk in TBST for 1 h at room temperature, then incubated overnight at 4 °C with mouse anti-Flag monoclonal antibody (1:5000, Abmart) and mouse anti-GAPDH antibody (1:50,000, Proteintech, Rosemont, IL, USA). After washing, membranes were incubated with HRP-conjugated goat anti-mouse secondary antibody (1:5000, Jackson ImmunoResearch, West Grove, PA, USA) for 1 h at room temperature. Signals were visualized using an enhanced chemiluminescence (ECL) detection system.

### 2.8. Structure Analysis

The impact of c.670_673dupGGTA on protein translation was predicted using Mutalyzer 3.0 (https://mutalyzer.nl/, accessed on 1 June 2025). Structural modeling of WT and mutant POU3F4 was performed via SWISS-MODEL (https://swissmodel.expasy.org/, accessed on 28 June 2025) using automated homology modeling, with the Golden hamster POU3F4 identified as the top structural homolog. Mutant models incorporated the frameshift-induced truncation predicted by Mutalyzer.

### 2.9. Statistical Analysis

Data are presented as the mean ± standard error of the mean (SEM). All experiments were performed as three independent biological replicates. Statistical analyses were conducted using GraphPad Prism 9.0 (GraphPad Software, San Diego, CA, USA). For comparisons between two groups, unpaired two-tailed Student’s t-test was used. For multiple comparisons, one-way analysis of variance (ANOVA) followed by Tukey’s post hoc test was applied. A *p*-value < 0.05 was considered statistically significant. Exact *p*-values are provided for all significant results.

## 3. Results

### 3.1. Clinical Features and Identification of a Novel Pathogenic Frameshift Variant in POU3F4

**Figure 1:**
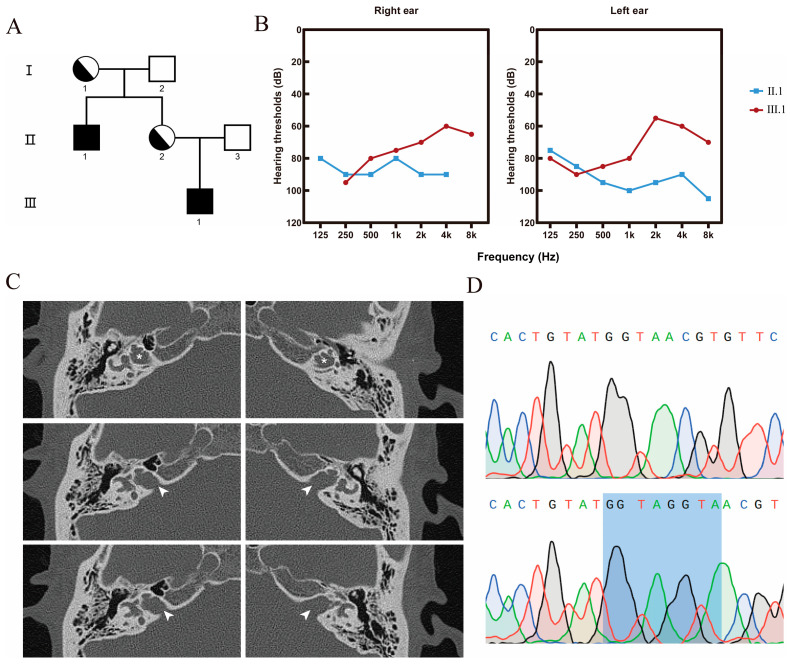
Identification of a *POU3F4* frameshift mutation in a Chinese family with X-linked deafness (DFNX2). (**A**) Pedigree of the three-generation Chinese family. Roman numerals (I, II, III) indicate generations, and Arabic numerals (1, 2, 3) indicate individuals within each generation. (**B**) Pure-tone air conduction audiograms of II.1 and III.1. (**C**) High-resolution temporal bone CT images of the proband (II.1) reveal bilateral inner ear malformations characteristic of DFNX2, including dilated internal auditory canals (arrowheads), absent modiolus (asterisks), and enlargement of the right vestibular aqueduct. (**D**) Sanger sequencing chromatograms showing the *POU3F4* c.670_673dupGGTA mutation. The duplicated “GGTA” sequence is highlighted in blue.

A three-generation Chinese family with X-linked non-syndromic hearing loss was enrolled in this study (Figure 1A), with two male individuals (II.1, the proband; and III.1, the proband’s nephew) affected by bilateral hearing loss. The proband (II.1), a 36-year-old male, had a history of progressive bilateral mixed hearing loss (Figure 1B). HRCT of the temporal bone revealed bilateral cochlear incomplete partition type III (IP-III) malformations, characterized by bilateral dilation of the IACs with lamina cribrosa deficiency, complete absence of the cochlear modiolus, mildly dilated vestibules with small inferior cystic outpouchings, mildly widened semicircular canals, and enlargement of the right vestibular aqueduct (Figure 1C). Notably, a prominent cerebrospinal fluid (CSF) gusher was encountered during the proband’s cochlear implantation surgery, which is a well-recognized surgical complication of the lamina cribrosa deficiency associated with *POU3F4* variants [13]. The younger affected individual (III.1), an 8-year-old male, displayed a congruent phenotypic profile, including profound mixed hearing loss since infancy and temporal bone CT confirmation of bilateral IP-III malformations with similar features.

Targeted NGS of the hereditary hearing loss gene panel identified three candidate variants: *GJB2* c.109G > A, *USH2A* c.2802T > G, and *POU3F4* c.670_673dupGGTA (Figure 1D). The first two are autosomal recessive variants present in a heterozygous state, which are generally insufficient to cause hearing loss in males unless they occur in a homozygous or compound heterozygous configuration [22,23]. The *POU3F4* duplication variant was hemizygous in the two affected males. Given the well-established role of *POU3F4* as an X-linked gene responsible for nonsyndromic hearing loss and the fact that the variant met the ACMG criteria for pathogenicity (PVS1 + PS3 + PM1 + PP3) [24], it was prioritized for further functional validation. Sanger sequencing confirmed the *POU3F4* c.670_673dupGGTA variant.

### 3.2. The p.(Asn225Argfs*2) Variant Alters the Subcellular Localization of POU3F4

**Figure 2:**
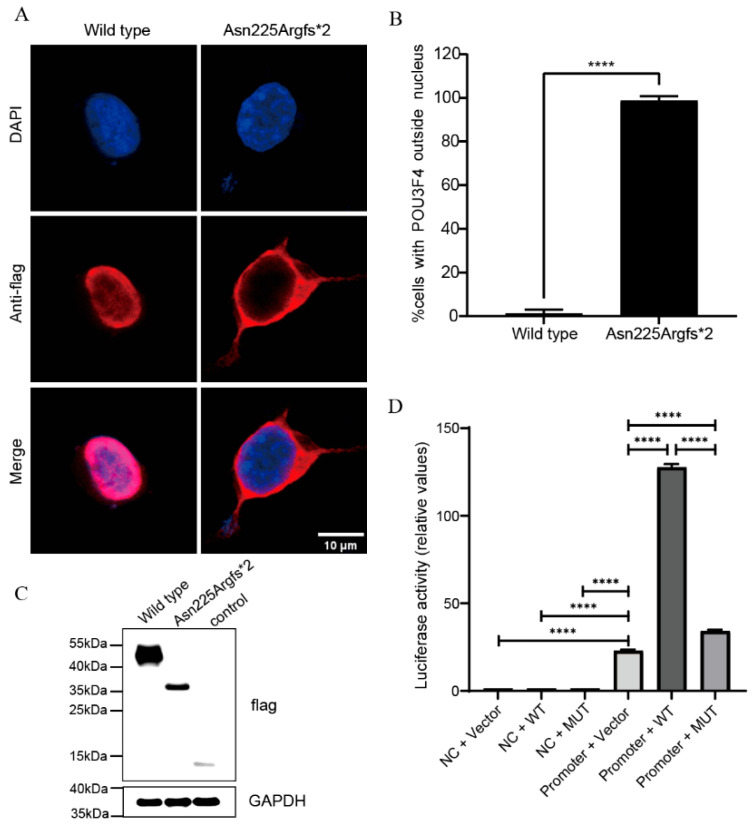
The p.(Asn225Argfs*2) mutation disrupts POU3F4 nuclear localization and transcriptional activity. (**A**) Representative confocal microscopy images of 293T cells transiently transfected with plasmids expressing WT or mutant POU3F4-3xFlag. (**B**) Quantitative analysis of subcellular localization. Data represent the percentage of transfected cells (*n* =100 per condition from three independent experiments) displaying cytoplasmic POU3F4-3xFlag signal. (**C**) Western blot of WT, mutant POU3F4-3xFlag and control proteins in 293T cells. GAPDH served as a loading control. (**D**) Dual-luciferase reporter assay assessing the effect of POU3F4 on its own promoter activity. **** *p* < 0.0001, NC: Promoter negative control, Vector: Empty expression vector, WT: *hPOU3F4-3xflag*, MUT: *hPOU3F4c. 670_673dupGGTA-3xflag*.

This variant is predicted to cause a frameshift starting at codon 225, resulting in an asparagine-to-arginine substitution and the introduction of a premature termination codon two amino acids downstream (p.(Asn225Argfs*2)). To determine whether the pathogenic effects of the identified mutation arise from aberrant subcellular localization, we examined the localization patterns of both WT and mutant proteins. Confocal microscopy revealed a significant redistribution: WT POU3F4 exhibited nuclear localization, whereas the p.(Asn225Argfs*2) mutant localized predominantly in the cytoplasm, with flag-tag being targeted as an indicator (Figure 2A).

### 3.3. The p.(Asn225Argfs*2) Variant Severely Impairs the Transcriptional Regulatory Activity of POU3F4

We next performed dual-luciferase reporter assays to evaluate the effect of the p.(Asn225Argfs*2) variant on the transcriptional activity of *POU3F4*, using its native promoter as the transcriptional target. The *POU3F4* promoter alone exhibited moderate intrinsic transcriptional activity, with a normalized Firefly/Renilla luciferase ratio of 22.92 ± 0.36. Co-transfection of the WT POU3F4 expression plasmid with the *POU3F4* promoter reporter resulted in a robust 5.6-fold increase in reporter activity (normalized ratio: 127.67 ± 1.07) relative to the promoter alone group. In marked contrast, co-transfection of the mutant POU3F4 expression plasmid led to a reduction in transactivation capacity, with relative activity falling to only 34.11 ± 0.45, which corresponds to approximately 27% of that observed for the WT protein (**** *p* < 0.0001; Figure 2D).

### 3.4. Western Blot Analysis of WT and Mutant POU3F4 Proteins

To determine whether the frameshift mutation affects protein stability, we performed Western blotting using an anti-flag antibody. As shown in Figure 2C, the wild-type POU3F4-3xFlag protein was robustly expressed as a specific band migrating between 40 and 55 kDa. The mutant p.(Asn225Argfs*2) protein also showed a clear band, with moderate expression intensity, migrating between 35 and 40 kDa. The negative control (empty vector) showed a weak signal near 15 kDa. These results demonstrate that the mutant protein is stably expressed and not subjected to rapid degradation, confirming that the observed functional defects are not due to loss of protein expression. The lower expression level may reflect mild instability or reduced translation efficiency but is not sufficient to explain the near-complete loss of transactivation capacity.

### 3.5. Structural Analysis of the Wild-Type and Mutant POU3F4 Proteins

**Figure 3:**
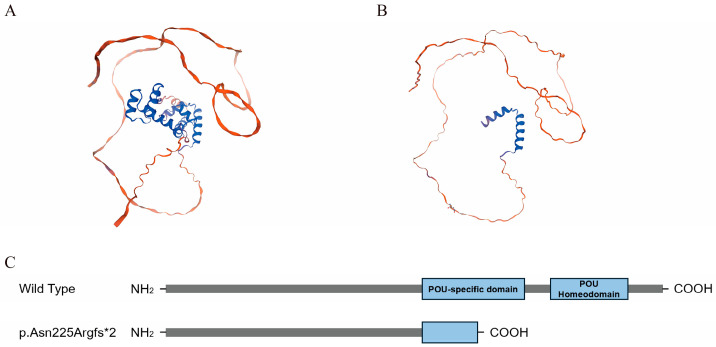
Structural modeling and conservation analysis of WT and mutant POU3F4. (**A**,**B**) Homology models of wild-type (**A**) and mutant (**B**) POU3F4. (**C**) Schematic diagram of wild-type and mutant protein products.

Homology modeling was performed to predict the structural consequences of the p.(Asn225Argfs*2) variant on POU3F4 protein structure. The variant results in a premature truncation within the POUS domain (residues 186–260). Furthermore, as the truncation occurs upstream of the entire POUH domain (residues 284–343), the mutant protein is completely devoid of both the POUS and POUH subdomains (Figure 3A,B), which are both required for the full DNA-binding and transcriptional regulatory activity of POU3F4. A schematic diagram of the domain structure of WT and mutant POU3F4 is shown in Figure 3C.

## 4. Discussion

This study identifies and functionally characterizes a novel frameshift variant in *POU3F4* (NM_000307.3; c.670_673dupGGTA, p.(Asn225Argfs*2)) in a three-generation Chinese family with classic clinical and radiological features of DFNX2. Unlike the vast majority of previously reported *POU3F4* variants, for which pathogenicity has been inferred solely from population frequency or prediction algorithms (e.g., SIFT, PolyPhen) without experimental validation [25], our study provides direct functional evidence. We comprehensively characterized the novel p.(Asn225Argfs*2) variant using subcellular localization, protein stability, and an autoregulatory luciferase reporter assay—an approach that remains rare in the field due to the lack of well-established physiological transcriptional targets.

The p.(Asn225Argfs*2) mutation results in truncation at residue 226, eliminating the entire C-terminal POUH and a portion of the upstream POUS. This structural disruption compromises POU3F4 function, as the intact bipartite POU domain is essential for its role as a sequence-specific transcription factor [21,26]. Confocal microscopy revealed cytoplasmic mislocalization of the mutant, contrasting with nuclear localization of WT POU3F4-3xflag. This mislocalization likely results from loss of a nuclear localization signal (NLS) embedded within an intact POUH [21]. Consequently, the mutant protein is unable to access nuclear target genes, thereby abrogating its transcriptional regulatory function.

Functional impairment was quantitatively confirmed by dual-luciferase reporter assays. WT POU3F4 robustly transactivated its own promoter, whereas the mutant retained only 27% of this activity. Structural analysis indicates that the mutation results in deletion of the C-terminal α3 and α4 helices of the POUS and the entire POUH domain. This extensive structural loss is predicted to severely compromise DNA-binding capacity [27]. The POUS α3 helix is essential for major-groove recognition [26]. Moreover, complete ablation of the POUH domain eliminates its intrinsic DNA-binding module, including its HTH motif responsible for recognizing the 5′-AAAT-3′ half-site [28]. Consequently, this mutation ablates the ability to form the hairpin structure, which requires the cooperative interaction of both intact POUS and POUH subdomains bound to their respective DNA half-sites [14]. Thus, these findings demonstrate that the mutation disables both the nuclear access and the intrinsic DNA-binding machinery essential for POU3F4-mediated transcriptional regulation. The Western blot results further support the conclusion that the mutant protein is expressed, excluding the possibility that the loss of function is solely due to nonsense-mediated mRNA decay or rapid protein degradation.

The clinical phenotype aligns precisely with the functional deficit. Both hemizygous males exhibited profound mixed hearing loss from early childhood, bilateral IP-III malformations on CT, and the pathognomonic perilymphatic “gusher” during surgery in II.1 [29,30]. These malformations are direct consequences of disrupted POU3F4 function during otic capsule development. POU3F4 expression in the otic mesenchyme is crucial for patterning the temporal bone, particularly formation of the modiolus and lamina cribrosa that separate the cochlea from the IAC [16,31]. Its loss leads to the defective partitioning (IP-III) and fistula causing CSF gushers. Furthermore, the profound sensorineural component likely stem from impaired spiral ganglion neuron survival, axon guidance, and gap-junction assembly essential for endocochlear potential maintenance [16]. Our findings are consistent with previous studies indicating that truncations within the POU domains consistently lead to mislocalization and complete loss of function, thereby correlating with severe phenotypes [32,33].

Beyond the direct effects on nuclear localization and DNA binding, POU3F4 is known to modulate a regulatory network of downstream targets indispensable for inner ear development, including *SLC6A20*, which encodes a sodium-dependent imino transporter critical for inner ear ion homeostasis [5]. Impaired transcriptional activity of POU3F4 is expected to dysregulate such targets, contributing to the developmental malformations and hearing loss in DFNX2 patients. Future studies should investigate the effect of this variant on these downstream targets.

Several limitations of the present study should also be acknowledged. First, it is based on a single family with two affected individuals, which limits the generalizability of the findings. Second, functional experiments were performed in 293T cells, which are not derived from inner ear tissue. These cells may lack the specific cofactors and regulatory mechanisms present in otic mesenchyme cells, which could affect POU3F4 function. Third, while we examined subcellular localization, protein stability, and autoregulatory transcriptional activity, we did not perform rescue experiments or validate effects on downstream targets; direct assessment of DNA-binding capacity by EMSA or ChIP was not conducted. Finally, structural predictions of DNA-binding loss, while strongly supported by domain analysis, await experimental confirmation in future studies. Despite these limitations, the main conclusions of this study remain valid and provide a foundation for further mechanistic exploration.

## 5. Conclusions

In conclusion, this study identifies and characterizes a novel frameshift mutation in *POU3F4*. Integrated functional and structural analyses demonstrate that this mutation compromises nuclear localization and eliminates DNA-binding capacity, resulting in loss of transcriptional activity. This molecular defect underlies the characteristic DFNX2 phenotype, underscoring the essential role of an intact C-terminal POU domain. These findings expand the mutational spectrum of *POU3F4*, reinforce genotype–phenotype correlations, and provide important insights for the diagnosis, genetic counseling, and management of X-linked deafness.

## Data Availability

The data underlying this article will be shared on reasonable request to the corresponding author.

## Supplementary Materials

The following supporting information can be downloaded at: https://www.mdpi.com/article/10.3390/life16060868/s1, Figure S1: Uncropped Western blot; Table S1: List of 127 deafness-related genes included in the targeted NGS panel; Table S2: Primers used for plasmid construction.

## Abbreviations

The following abbreviations are used in this manuscript:

SNHL	sensorineural hearing loss
NSHL	non-syndromic hearing loss
IAC	internal auditory canal
POUS	POU-specific domain
POUH	POU homeodomain
HTH	helix-turn-helix
NGS	next-generation sequencing
WT	wild-type
CSF	cerebrospinal fluid
