# Supplementary material for: Identification and Functional Characterization of a Novel POU3F4 Frameshift Mutation in a Chinese Family
Source: Life (Basel). 2026 May 22;16(6):868. doi: 10.3390/life16060868 (PMC13302508; doi:10.3390/life16060868)
Supplement: Supplementary file 1 [file life-16-00868-s001.zip › Table S1.pdf]

Supplementary Table S1: Primers used for plasmid construction

PC-h-POU3F4-F1	GTTAAGCTTGGTACCGAGCTCGGATCCGCCACCAtgGACTACAAAGACCATGACGGTGATTATAAAGATCAT
PC-h-POU3F4-R1	ggattcgaggcagctgtggcCTTGTCATCGTCATCCTTGTAATCGATGTCATGATCTTTATAATCACCGTC
PC-h-POU3F4-F2	ACAAGgccacagctgcctcgaatccctacagcattctcagtt
PC-h-POU3F4-R2	GCCACTGTGCTGGATATCTGCAGAATTCtcagagatcatggcaagatgtgtc
PC-h-POU3F4 c670-F1	GTTAAGCTTGGTACCGAGCTCGGATCCGCCACCAtgGACTACAAAGACCATGACGGTGATTATAAAGATCAT
PC-h-POU3F4 c670-R1	ggattcgaggcagctgtggcCTTGTCATCGTCATCCTTGTAATCGATGTCATGATCTTTATAATCACCGTC
PC-h-POU3F4 c670-F2	ACAAGgccacagctgcctcgaatccctacagcattctcagtt
PC-h-POU3F4 c670-R2	aacacgtTACCtaccatacagtgtgcccagcgccaaccccac
PC-h-POU3F4 c670-F3	cactgtatggtaGGTAacgtgttctcgcagaccaccatctgcag
PC-h-POU3F4 c670-R3	GCCACTGTGCTGGATATCTGCAGAATTCtcagagatcatggcaagatgtgtc
