# Supplementary material for: Identification and Functional Characterization of a Novel POU3F4 Frameshift Mutation in a Chinese Family
Source: Life (Basel). 2026 May 22;16(6):868. doi: 10.3390/life16060868 (PMC13302508; doi:10.3390/life16060868)
Supplement: Supplementary file 1 [file life-16-00868-s001.zip › Table S2.pdf]

Table S2: List of 127 deafness related genes included in the targeted NGS panel.

Types of Hereditary Hearing Impairment	Genes
Autosomal recessive nonsyndromic hearing impairment	*GJB2, GJB6, MYO7A, MYO15A, FOXI1, KCNJ10, SLC26A4, TMIE, TMC1, TMPRSS3, OTOF, CDH23, ATP2B2, GIPC3, STRC, OTOG, USH1C, TECTA, OTOA, PCDH15, RDX, GRXCR1, TRIOBP, CLDN14, MYO3A, DFNB31, ESRRB, ESPN, MYO6, GJA1, HGF, ILDR1, MARVELD2, MPZL2, DFNB59, SLC26A5, LRTOMT, LHFPL5, BSND, MSRB3, LOXHD1, TPRN, GPSM2, PTPRQ, SERPINB6, GJB3*
X-link hereditary hearing impairment	*PRPS1, POU3F4, SMPX*
Syndromic hearing impairment	*SERAC1, PDSS1, FGFR3, FGFR1, FGFR2, PHEX, DLX5, TNFRSF11B, COL2A1, COL11A1, COL9A1, COL9A2, COL4A3, COL4A4, COL4A5, BSND, SOX9, PAX2, GATA3, SLC19A2, IGF1, PAX3, MITF, SNAI2, EDNRB, EDN3, SOX10, HOXA1, SOBP, EYA1, SIX5, SIX1, CHD7, SEMA3E, SMAD4, FGF3, TCOF1, PRRX1, GLI3, HOXA2, KCNQ1, KCNE1, CACNA1D, ALMS1, LRP2, TIMM8A, NDP, WFS1, OPA1, SLC4A11, MYO7A, USH1C, CDH23, PCDH15, USH1G, USH2A, GPR98, PDZD7, DFNB31, CLRN1, MT-TK, MT-TE, MT-TL1, SLC26A4, KCNJ10, FOXI1*
Autosomal dominant nonsyndromic hearing impairment	*ACTG1, CCDC50, CEACAM16, COCH, CRYM, DFNA5, DIABLO, DIAPH1, DSPP, EYA4, GJB2, GJB3, GJB6, GRHL2, KCNQ4, MYH14, MYH9, MYO1A, MYO6, MYO7A, POU4F3, SIX1, SLC17A8, TECTA, TJP2, TMC1, WFS1, DIAPH3*
Maternally inherited hearing impairment	*MT-RNR1, MT-TS1*
