# Supplementary figures and images for: Identification and Functional Characterization of a Novel POU3F4 Frameshift Mutation in a Chinese Family
Source: Life (Basel). 2026 May 22;16(6):868. doi: 10.3390/life16060868 (PMC13302508; doi:10.3390/life16060868)

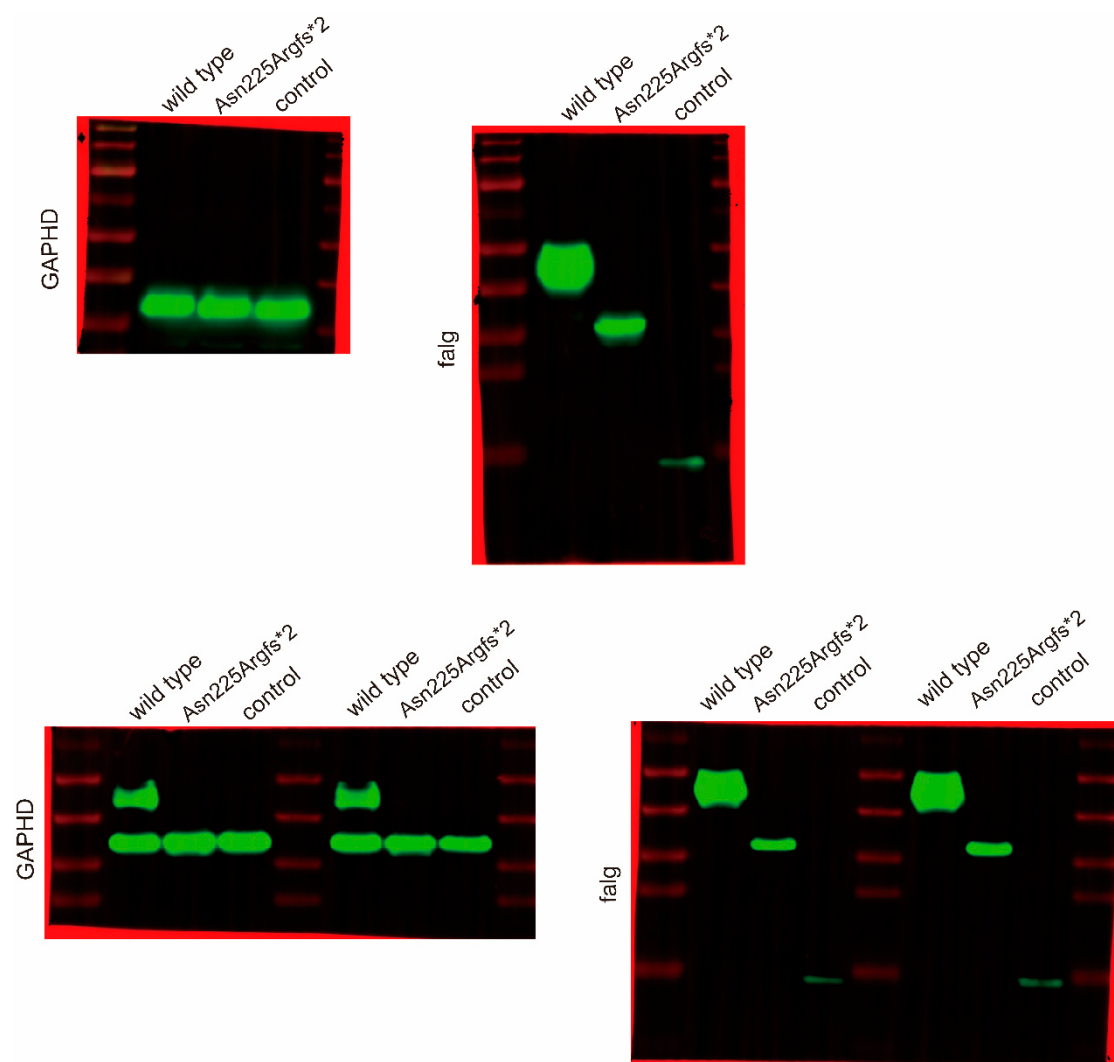

Figure S1: Uncropped Western blot

Supplement: Supplementary file 1 [file life-16-00868-s001.zip › Figure S1.pdf]
